# Systematic review of economic evaluations of aromatase inhibitors in estrogen receptor-positive breast cancer: quality evaluation

**DOI:** 10.1186/s12913-023-09432-5

**Published:** 2023-06-26

**Authors:** Maha F. Althuwaibi, Cristina Fernandez-Garcia, Louise Hayes, Richard McNally, Diarmuid Coughlan

**Affiliations:** 1 College of Pharmacy, Princess Nourah Bint Abdulrahman University, Riyadh, 11671, Saudi Arabia; 2 Population Health Sciences Institute, Newcastle University, Newcastle upon Tyne, United Kingdom

**Keywords:** Aromatase inhibitors, Tamoxifen, Cost-effectiveness, Systematic review, Breast cancer, Quality

## Abstract

**Background:**

Breast cancer (BC) is a leading cause of premature death in women and the most expensive malignancy to treat. Since the introduction of targeted therapies has resulted in changes to BC therapy practices, health economic evaluations have become more important in this area. Taking generic medications, Aromatase Inhibitors (AIs), as a case study, we conducted a systematic review of the recent economic evaluations of AIs for estrogen receptor-positive breast cancer patients and evaluated the quality of these health economic studies.

**Objective:**

To systematically review and examine the quality of the available economic studies of AIs in estrogen receptor-positive breast cancer.

**Methods:**

A literature search was performed using six relevant databases **(**MEDLINE, Embase, Database of Abstracts of Reviews of Effects, Health Technology Assessment Database, NHS Economic Evaluation Database, and SCOPUS) from January 2010 to July 2021. All economic studies were independently assessed by two reviewers using the Consolidated Health Economic Evaluation Reporting Standards (CHEERS) checklist to evaluate the quality of the economic evaluations. This systematic review is registered in the PROSPERO database. To compare the different currencies used in these studies, all costs were converted to international dollars (2021).

**Results:**

A total of eight studies were included in the review; six (75%) were performed from the healthcare providers’ perspective. They were conducted in seven different countries, and all were model-based analyses using Markov models. Six (75%) considered both Quality Adjusted Life Years (QALYs) and Life Years (LY) outcomes, and all costs were derived from national databases. When compared to tamoxifen, AIs were generally cost-effective in postmenopausal women. Only half of the studies addressed the increased mortality following adverse events, and none mentioned medication adherence. For the quality assessment, six studies fulfilled 85% of the CHEERS checklist requirements and are deemed good quality.

**Conclusion:**

AIs are generally considered cost-effective compared to tamoxifen in estrogen receptor-positive breast cancer. The overall quality of the included studies was between high and average but characterizing heterogeneity, and distributional effects should be considered in any future economic evaluation studies of AIs. Studies should include adherence and adverse effects profiles to provide evidence to facilitate decision-making among policymakers.

**Supplementary Information:**

The online version contains supplementary material available at 10.1186/s12913-023-09432-5.

## Highlights

- This is a systematic review looking at Aromatase Inhibitors (AIs) in breast cancer since 2010.
- Overall, the studies included were of high and average quality.
- The reporting of the models has improved.
- The structural uncertainty is still underreported and has not incorporated issues such as medication adherence and side-effect profile.

**Supplementary Information:**

The online version contains supplementary material available at 10.1186/s12913-023-09432-5.

## Background

Breast cancer (BC) is the most diagnosed cancer in women globally. In 2020, there were an estimated 2.3 million new cases of BC and 685,000 deaths from BC worldwide [1]. It is the leading cause of cancer death in women in developing countries [1]. The high incidence and prevalence of BC impose a tremendous financial burden and carry huge socioeconomic, emotional, and public health implications. Policymakers need robust evidence on the cost-effectiveness of different treatment options to base decisions on how best to use scarce healthcare resources.

BC treatment options are determined based on the tumor’s type, stage, and grade and whether it is sensitive to hormones. Hormonal therapy is the cornerstone of adjuvant systemic treatment for patients with hormone receptor-positive BC [2]. Aromatase inhibitors (AIs) (such as letrozole, anastrozole, and exemestane) and tamoxifen are hormonal therapies used in women with breast cancer. AIs reduce recurrence and cancer mortality rates by 30% and 15%, respectively, compared with tamoxifen [3].

Several pertinent systematic reviews of economic evaluations of AIs for the treatment of hormone receptor-positive BC have been published. John-Baptiste et al. (2013) concluded that studies were overestimating the cost-effectiveness of AIs and recommended being cautious when drawing conclusions about the value of AIs versus tamoxifen [4]. Frederix et al. (2012) concluded that the included studies in their review did not demonstrate if AIs represent better value for money than tamoxifen [5]. Diaby et al. (2015) recommended additional studies to elucidate the cost-effectiveness of AIs versus tamoxifen in early-stage breast cancer [6].

Previous systematic reviews were conducted before the availability of AIs in generic formulations (patent expiration in 2011) and before long-term follow-up studies of AIs were available. This would affect the cost-effectiveness analysis of AIs due to the availability of lower cost generic drugs. Furthermore, a new checklist, Consolidated Health Economic Evaluation Reporting Standards (CHEERS), to optimize reporting of health economic evaluations was published in 2013 and updated in 2022 [7]. In addition, previous systematic reviews did not evaluate the structural uncertainty.

Economic evaluation is an essential part of the health technology assessment (HTA) process to help inform healthcare decision-makers. The quality of these studies is crucial to countries with limited HTA resources. This review will help authors from such countries to improve the quality of their studies so that policymakers will have the tools to help them make better decisions. We systematically reviewed the economic evaluation of AIs since 2010, examined the quality of these studies, and summarized the evidence on drivers of cost-effectiveness. Our aim was to look at the model structures and the input parameters and how the analyses were conducted. A comparative analysis of model structure and parametrization using a checklist and guidelines for models was conducted to improve our understanding of the quality of current evidence.

## Methods

### Literature search

A comprehensive literature search for economic evaluations of AIs versus tamoxifen in females with Estrogen Receptor positive BC was performed using MEDLINE (July 16, 2021), Embase (2021 July 16), Cochrane library (Database of Abstracts of Reviews of Effects, Health Technology Assessment Database, and NHS Economic Evaluation Database), and SCOPUS (July 2021).

The electronic search strategy was based on (PICOS): Population (postmenopausal females with BC), Interventions (at least one AIs), Comparators (Tamoxifen), Outcomes (health outcomes such as Quality Adjusted Life Years (QALY) or Life Years Gained (LYG) or life years saved (LYS)) and Study designs (economic study, cost-effectiveness analysis (CEA), cost-utility analysis (CUA) or cost-benefit analysis (CBA)). The exclusion criteria were: (i) descriptive costing studies as they are not considered full economic evaluations, (ii) Conference abstracts because they lack details about the methods, and (iii) economic evaluation addressing extended adjuvant therapy. No language restrictions were imposed (See supplementary appendix S1 for search strategy).

### Study selection

The study selection procedure encompassed three main stages. The first stage was to import all the references to Endnote and remove duplicates. The second stage was to evaluate the remaining studies based on the title and abstract and studies that did not meet the inclusion criteria were excluded. In the third stage, the full articles of potentially relevant studies were retrieved, and those that met the inclusion criteria were included in the current review. Reviewer one (MF) and reviewer two (DC) screened the identified abstracts and full texts for eligibility.

### Data extraction

We extracted the characteristics of the identified studies in two tables. A summary of the pertinent study characteristics: publication year, country, perspective, type of model, type of economic evaluation, time horizon, sponsorship, discount rate, and currency were extracted along with a summary of the model characteristics: source of data, methods of measuring outcomes, included costs, AI, type of sensitivity analysis, incremental cost-effectiveness ratio (ICER), stage of BC, line of treatment, population, and conclusion.

To allow direct comparison across countries, all costs were converted to International Dollars and then inflated to reference year (2021) using the ‘CCEMG – EPPI-Centre Cost Converter’ (v.1.6 last update: April 29, 2019), a free web-based tool for adjusting estimates of cost expressed in one currency and price year to a specific target currency and price year [8]. Data were extracted using Microsoft Excel and performed by three assessors (MF, DC, LH), and we used the help of (CFG) in extracting the two articles in Spanish.

### Quality assessment

The Consolidated Health Economic Evaluation Reporting Standards (CHEERS) Statement was adopted to critically appraise the studies. The 28-item CHEERS checklist consists of 7 domains: Title (1 item); Abstract (1 item); Introduction (1 item); Methods (18 items); Results (4 items); Discussion (1 item); and Other relevant information (2 items) [7]. CHEERS checklist is not a scoring instrument, but we adopted the same tool based on other review studies indicating ‘yes’ when the criteria were met, ‘no’ when they were unfulfilled, and ‘‘not applicable’ when they were not required for that type of study. We divided the studies into three quality categories according to the proportion of items achieved: high (> 75%), average (50–75%) and poor (< 50%) [9].

The quality assessment was conducted by one assessor (MF) for all studies except Spanish studies, which were evaluated by (CFG), and ambiguities were resolved by consulting another assessor (DC). Sensitivity analysis was used to address uncertainty, which is divided into three categories: structural, methodological, and parameter.

#### Structural uncertainty

Structural uncertainty relates to whether all relevant processes are represented in the model. We abstracted the adverse events mentioned in the analysis and determined whether their effect on mortality was incorporated in the analysis or not.

#### Methodological uncertainty

Methodological uncertainty refers to choices about population, time horizon, and study perspective that impact how economic evaluation estimates are calculated. This includes sensitivity analysis (SA) for extrapolating beyond the follow-up time of studies, did the analysis address different subgroups such as older women, women at high risk of side effects (SE), women with comorbidities, and women at low risk of BC recurrence?

#### Parameter uncertainty

Parameter uncertainty concerns the numerical values of input parameters. We abstracted the data source on both BC recurrence and adverse event rates associated with AIs. Then, we determined whether the authors perform the following or not: SA on the risk of BC recurrence, SA on SE (including fracture, cardiovascular events, stroke, thromboembolic events, endometrial cancer), probabilistic sensitivity analysis (PSA), and value of information analysis (VOI) to critique the authors’ handling of parameter uncertainty.

## Results

### Literature search

**Fig. 1:**
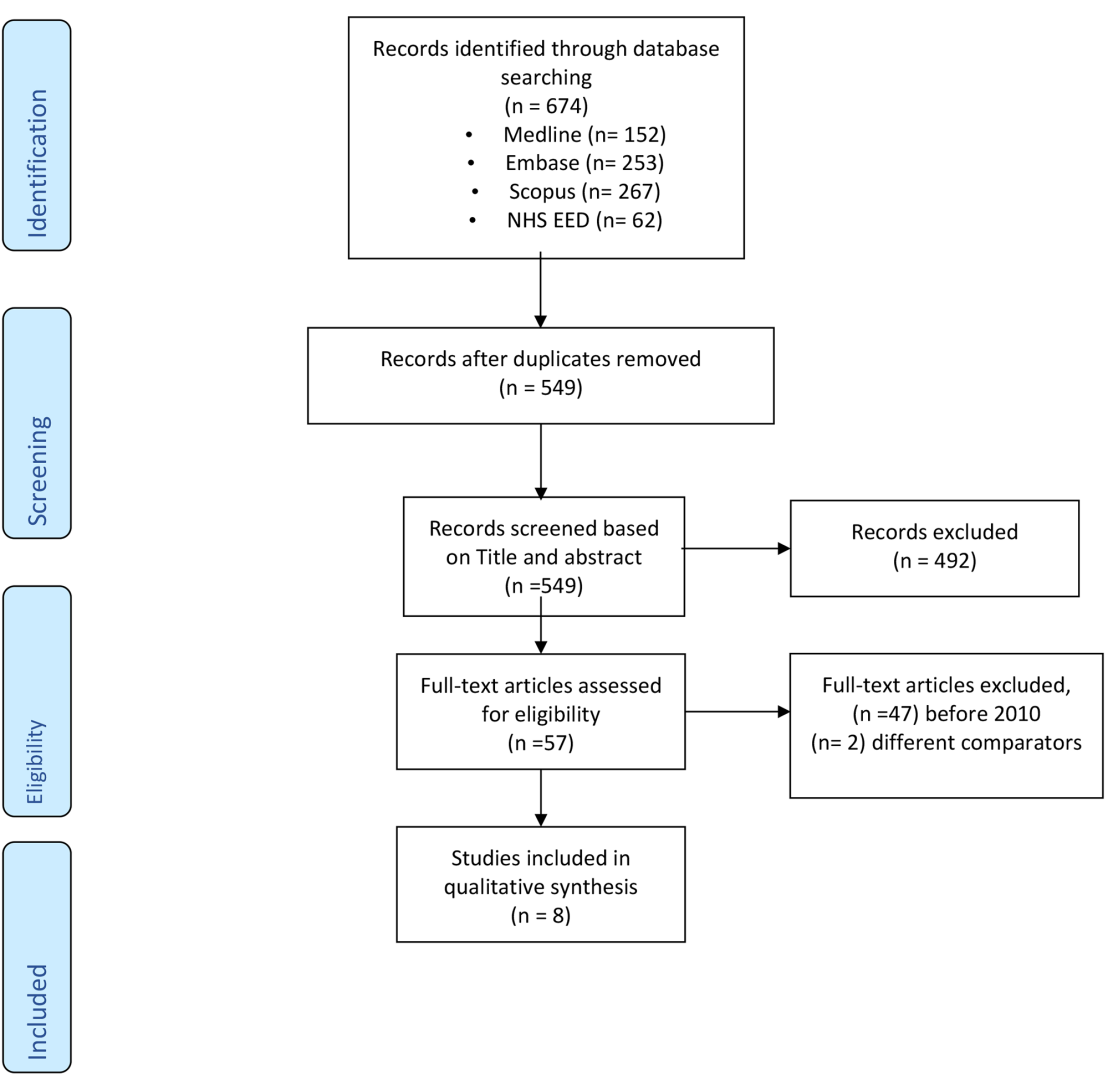
PRISMA flow diagram of included studies

Records identified through database synthesis were 734 references, among which 185 were duplicates, 492 were excluded after screening and analysis of titles and abstracts for not matching the eligibility criteria, 47 articles were excluded due to date restriction, and two were excluded because of the comparators. A total of eight papers were retrieved and analyzed [10–17]. (Fig. 1)

### Characteristics of studies included in the review

A total of eight articles were included in the final study, of which six were published in English [11–16] and two in Spanish [10, 17]. Studies were conducted in different countries including Mexico [10], China [11], Canada [12], Singapore [13], Germany [14, 15], Korea [16], and Colombia [17]. In the majority of the studies, authors conducted the analysis from the perspective of the health care system (n = 6; 75%) [10–12, 14, 15, 17]; only two studies were conducted from a societal perspective [13, 16]. 75% of studies (*n* = 6) considered both QALY and LY outcomes [11–16], while the remaining two studies used recurrence rate [10] and overall survival [17] as outcome measures. All economic evaluations involved were model-based analyses using Markov cohort models. All studies considered direct costs, except one study considered both direct and indirect costs [16]. Shih et al. involved direct costs only despite conducting their study from a societal perspective [13]. All studies clearly stated the price and currencies, and costs were derived from local sources and/or national databases. The publication years ranged from 2010 to 2018 (Table 1).

**Table 1:** Study characteristics

Study Country	Perspective/ Time Horizon	Type of Model/ economic evaluation	Aromatase Inhibitor	Population Studied (Age at entry)
Ye, M. et al[11] (2018) China	Chinese Healthcare system/ Lifetime	Markov CEA	Letrozole	Postmenopausal women with newly diagnosed early ER + ve BC after lumpectomy, 57 yrs (27–79 yrs)
Djalalov, S. et al[12] (2015) Canada	Canadian health system/ Lifetime	Markov CEA	Treated the AI drug class as a group without reference to a specific drug	Postmenopausal women with ER + ve early BC, 65 yrs
Shih, V.et al[13] (2012) Singapore	Societal/ Lifetime	Markov CEA and CUA	Anastrozole	Postmenopausal women with HR + ve early-stage BC who had completed primary therapy, 64 yrs
Mould-Quevedo[10] et al. (2011) Mexico	Healthcare payers/ 10 years	Markov CEA	Anastrozole Letrozole Exemestane	Postmenopausal HR + BC females. The cohort was divided into two groups. One for females with positive lymph nodes (LN+) and one for females with negative lymph nodes (LN-) NR
Lux, M. et al.[15] (2011) Germany	Healthcare system/ 20 years	Hybrid and Markov CBA*	Anastrozole Letrozole	Postmenopausal women with HR + ve BC, 76–80 yrs
Gamboa et al[17] (2010) Colombia	Colombian health care system/ 30 years	Markov CEA	Anastrozole	Postmenopausal women with ER + ve early BC, 50 yrs
Lee, et al[16] (2010) Korea	Societal/ 35 years	Markov CEA	Anastrozole Letrozole	Postmenopausal women with HR + ve early BC, 50 yrs
Lux, M. et al.[14] (2010) Germany	German healthcare system/ 25 years	Markov CEA	Anastrozole	Postmenopausal women with HR + ve early BC, 64 yrs

Key; CEA: Cost-Effectiveness Analysis, CBA: Cost- Benefit Analysis, CUA: Cost-Utility Analysis, ER: estrogen receptor, HR: hormone receptor, NR: not reported

*Paper title is CBA, but it is a CE

One study modeled for ten years [10], while most studies used a lifetime horizon or ranged between 20 and 35 years. Discounting of costs was made in all studies; half of the studies used a 3% discount rate, and the other half used a 5% discount rate. Three studies [11, 14, 15] justify choosing this discount rate, but others did not. Most studies compared one AI vs. Tamoxifen (n = 4), one compared letrozole vs. tamoxifen [11] and three compared anastrozole vs. tamoxifen [13, 14, 17]. The remaining studies compared anastrozole or letrozole vs. tamoxifen [15, 16] (n = 2), one comparing the three AIs (anastrozole or letrozole or exemestane) vs. tamoxifen [10], and one treated the AI drug class as a group without reference to a specific drug [12]. Efficacy data were derived from the results of clinical trials or literature. (Table 2)

**Table 2:** Model Characteristics

Author	Source of Data	Outcomes	Type of sensitivity analysis	ICER conversion to I$ 2021	Findings
Ye, M. et al. [11]	**Effectiveness**: published randomized clinical trials meta-analyses (EBCTCG). **Costs**: from published Chinese studies.	- Progression-free LY’s - Overall LY’s - QALYs	- PSA (second-order Monte Carlo technique) - One-way sensitivity analyses	11,510/QALY	Adjuvant endocrine therapy with Letrozole is a cost-effective strategy compared to tamoxifen in women with early BC
Djalalov, S.et al [12]	**Effectiveness**: Medical literature, meta-analysis (BIG 1–98 trial and ATAC trial) **Costs**: Ontario Ministry of Health and Long-Term Care, Ontario Drug Benefit Formulary Costs, published Canadian studies.	QALY’s (Utility weights)	- PSA (Monte Carlo simulation) - Deterministic sensitivity analysis	NR	In postmenopausal women with ER + ve early BC, strategies using AIs appear to provide more benefit than strategies using TAM alone. Sequential strategies using TAM and an AI appear to provide benefits similar to those provided by upfront AI but at lower cost
Shih, et al [13]	**Effectiveness**: ATAC trial, interviews with oncology nurses, local financial electronic databases, published literature **Costs**: were obtained via financial electronic databases of the NCCS and the Singapore General Hospital	- Cost per LY survival - Cost per QALY gained	Multiple one-way sensetivity analyses	ICER of anastrozole was: − 242,815/ LY − 133,536 / QALY gained	If the WHO recommendation of 1 to 3x GDP range is an acceptable threshold, anastrozole is deemed cost-effective compared with tamoxifen in the treatment of early-stage BC
Mould-Quevedo,et al [10]	**Effectiveness**: Probabilities derived from published data. **Costs**: obtained from the Mexican Social Security Institute	- Non- recurrence rate - Time to recurrence	PSA (2nd order Monte Carlo simulation)	NR	Sequential treatment with tamoxifen/ exemestane appeared to be a cost-effective alternative among the therapies, which includes an aromatase inhibitor for women with BC in Mexico
Lux, M. et al. [15]	**Effectiveness**: BIG 1–98 study, ATAC study, and EBCTG study **Costs**: generic prices	- Recurrence rate - Overall survival - QALY (Utility weights)	PSA (Monte Carlo simulation with 2000 scenarios).	- ICER for anastrozole is 206,256 /QALY - ICER for letrozole is 45,019/QALY	The present model, including the inverse probability of censoring weighted analysis (IPWC) for letrozole and generic prices for both AIs shows that letrozole is cost-effective.
Gamboa, et al [17]	**Effectiveness**: Literature **Costs**: Treatment and adverse events costs derived from information provided by several health service providers over a period of 12 months. Relapse costs based on the individual costs for 23 women provided by the National Institute of Cancer	- Survival - Time free from disease	- PSA - One- way sensitivity analysis	- Non-discounted ICER = 29.51 /LY - Discounted ICER = 40.35/ LY	Compared to tamoxifen, adjuvant therapy with anastrozole yields an additional 0.49 disease-free years. The additional cost per disease-free year gained is 37,071 Colombian pesos. Tamoxifen has a higher probability of being cost-effective at all WTP points considered in the analysis
Lee, et al [16]	**Effectiveness**: published studies (EBCTCG meta-analysis, the ATAC trial, and the BIG 1–98 trial) **Costs**: Drug costs were based on the 2009 pharmaceutical prices that were weighted by the prescription volume, which was issued by the Korean Health Insurance Review and Assessment Service (HIRA) in the first half year of 2009	- QALY’s - LY	Deterministic sensitivity analysis	- for anastrozole 31,858 - for letrozole 29,791	Anastrozole and letrozole were both cost-effective treatments compared to tamoxifen. When anastrozole and letrozole were compared indirectly in the overall population, their cost-effectiveness ratios were too similar to decide which treatment was superior to the other When the population was divided by nodal status, anastrozole was more cost-effective than letrozole in the node-negative group, and letrozole was more effective in the node-positive group
Lux, M. P. et al. [14]	**Effectiveness**: published literature and expert opinion (ATAC trial) **Costs**: costs were derived from standard sources.	- QALY - Overall survival	- Scenario analyses - Deterministic sensitivity analysis - PSA	for anastrozole compared to tamoxifen was 32,616/QALY gained	Adjuvant treatment with anastrozole for postmenopausal women with HR + EBC is a cost-effective alternative to tamoxifen

Key: EBCTCG: Early Breast Cancer Trialists’ Collaborative Group, ICER: incremental cost-effectiveness ratio, I$: International dollars, ATAC: The Arimidex, Tamoxifen Alone or in Combination trial, BIG 1–98: The Breast International Group 1–98 trial, QALY: quality-adjusted life years, LY: life year, PSA: probabilistic sensitivity analysis, NR: not reported

Most studies report an ICER value except for two that didn’t calculate the ICER [10, 12]. The ICER values for anastrozole and letrozole after conversion to 2021 International dollars ranged between $ 40 to $ 206,256/QALY and $ 11,510 to $ 45,019/ QALY, respectively.

The Markov cycle length used in all studies was yearly except for two studies that used a 1-month [11] and a 3-month cycle [14]. Most of the studies (n = 6) concluded that when compared to tamoxifen, AIs were cost-effective at a commonly accepted threshold for cost-effectiveness (less than $50k /QALY [18]), except for two studies [10, 17] which concluded that tamoxifen is cost-effective.

### Quality assessment

The quality assessment results using the CHEERS checklist per study are summarized in (Table 3). The mean number of fulfilled criteria for the CHEERS checklist was 22 out of 28. The most frequent partially or not reported items were health economic analysis plan (item 4), characterizing heterogeneity (item 18), characterizing distributional effects (item 19), approach to engagement with patients and others affected by the study (item 21), and effect of engagement with patients and others affected by the study (item 25).

The quality of the included studies is between high and average levels; 75% of the studies (n = 6) were of average quality, and 25% (n = 2) were of high quality according to our criteria.

**Table 3:** quality assessment of Cost-effectiveness studies

			Ye, M.et al [11]		Djalalov, S.et al [12]		Shih, et al. [13]		Mould-Quevedo,et al. [10]		Lux, M.et al [15]		Gamboa,et al. [17]		Lee, et al. [16]		Lux, M.et al [15]	
		Title																
1		Title	√		√		√		√		√		√		√		√	
		Abstract																
2		Abstract	√		√		√		√		√		√		x		√	
		Introduction																
3		Background and objectives	√		√		√		√		√		√		√		√	
		Methods																
4		Health economic analysis plan	x		x		x		x		x		x		x		x	
5		Study population	√		√		√		√		√		√		√		√	
6		Setting and location	√		√		√		√		√		x		√		√	
7		Comparators	√		√		√		√		√		√		√		√	
8		Perspective	√		x		√		√		√		√		√		√	
9		Time horizon	√		√		√		√		√		√		√		√	
10		Discount rate	√		√		√		√		√		√		√		√	
11		Selection of outcomes	√		√		√		√		√		√		√		√	
12		Measurement of outcomes	√		√		√		x		√		√		√		√	
13		valuation of outcomes	√		√		√		x		√		x		√		√	
14		Measurement and valuation of resources and costs	√		√		√		√		√		√		√		√	
15		Currency, price date, and conversion	√		√		√		√		√		√		√		√	
16		*Rationale and description of model*	√		√		√		√		√		√		√		√	
17		Analytics and assumptions	√		√		√		√		√		√		√		√	
18		Characterizing heterogeneity	√		x		√		x		x		√		√		x	
19		Characterizing distributional effects	x		x		x		x		x		x		x		x	
20		Characterizing uncertainty	√		√		√		√		√		√		√		√	
21		Approach to engagement with patients and others affected by the study	x		x		x		x		x		x		x		x	
		Results																
22		Study parameters	√		√		√		√		√		√		√		√	
23		Summary of main results	√		√		√		√		√		√		√		√	
24		Effect of uncertainty	√		√		√		√		√		√		√		√	
25		*Effect of* engagement with patients and others affected by the study	√		x		x		x		x		x		x		x	
		Discussion																
26		Study findings, limitations, generalizability, and current knowledge	√		√		√		√		√		√		√		√	
		Other Relevant Information																
27		Source of funding	√		√		√		√		√		√		x		√	
28		Conflicts of interest	√		√		√		√		x		√		x		x	

#### Data sources

All the studie’s authors used one or two RCTs as a source of data to estimate the impact of hormonal therapies on breast cancer recurrence. Most data were taken from either the ATAC trial (Arimidex or Tamoxifen Alone or in combination trial) [19] and/or the BIG 1–98 trial (the breast international group trial) [20].

Costs were obtained from national databases; Ye et al. and Djalalov et al. are the only two studies that mentioned using the generic costs of the drugs [11, 12].

#### Handling structural uncertainty

Half of the studies (n = 4, 50%) addressed the increased mortality following adverse events (Table S1).

#### Handling methodological uncertainty

Few economic evaluations performed sub-group analyses to address patient heterogeneity related to older women (n = 2, 25%), and no study looked at women at low risk of breast cancer recurrence. A large proportion (75%) did not assess the impact of uncertainty arising from extrapolating beyond the trial data. Five studies (62.5%) vary the discount rate in the sensitivity analysis (Table S1).

#### Handling parameter uncertainty

All the studies reported sensitivity analysis on the risk of breast cancer recurrences. Two studies did not perform sensitivity analysis on the risk of adverse events (n = 2, 25%). Six studies (75%) performed PSA. One study conducted a VOI analysis. (Table S2)

Detailed information on the handling of parameter, structural and methodological uncertainty are available in (Tables S1 and S2).

## Discussion

In this study, we systematically reviewed and assessed the quality of eight economic evaluations comparing AIs to tamoxifen for early-stage breast cancer published between 2010 and 2021, covering the perspectives of Chinese, Korean, German, Canadian, Singapore, Colombian and Mexican healthcare systems. When compared to tamoxifen, AIs were reported to be cost-effective in postmenopausal women with early-stage BC in most studies (75%) at a commonly accepted threshold for cost-effectiveness (less than $50k /QALY).

Two previous systematic reviews of economic evaluations conducted on AIs and tamoxifen in early-stage BC. John-Baptiste et al [4]. identified 18 cost-effectiveness studies between 2004 and 2010, while Frederix et al [5]. analyzed 20 articles about the cost-effectiveness of endocrine treatments published between 2000 and 2010. These reviews concluded that there is an overestimation of the cost-effectiveness of AIs, and there is a need for standardized models to help in decision-making. Our study now finds that AIs are cost-effective based on high to average-quality study methodology. The general evaluation approaches in all studies had a significant degree of similarity. First, all the evaluations used a Markov model. Secondly, all studies used an RCT as a source of data and national costs. But despite this fact, the reported cost-effectiveness results were not consistent across all the evaluations; this variation could be due to the difference in treatment costs in different countries.

In two studies conducted by Lux et al [14]. there were considerable differences in ICER $ 32,616/QALY and $ 206,256/QALY even though they were conducted from the German healthcare perspective, using the same discount rate (3%), the same AI (anastrozole), and similar time horizon (20–25 yrs.) and only differ in age of the participant at entry (76–80 and 64 yrs.), the higher ICER was associated with using generic drug costs.

In studies comparing letrozole to tamoxifen, the lowest ICER was associated with using generic drug prices in the latest study (2018). The ICER of the two other studies was doubled; this could be due to different discount rates, different settings, different lifetime horizons, and different ages at entry. The study in Mexico used a short time horizon of 10 years which failed to capture the full costs and effects of chronic diseases [10]. The difference between studies in the participant’s age at entry should be considered; knowing the side effects of AIs and how they affect older ages could lead to differences in the costs of side effects in different age groups.

All studies except two used a yearly Markov model cycle length without justification; the recommended cycle length is 3 months because recurrences are very relevant for the outcome and using a 3-month cycle is a better representation of the course of the disease.

Regarding the quality of reporting these evaluations, we observed that the reporting was sufficient except for reporting sub-group analysis to address heterogeneity, increase mortality following adverse events, and approaches to engage patients or others affected by the study which were partially reported. We recognized that all the studies are not following any checklists to evaluate the quality of their studies, we highly recommend using checklists to improve the reporting and hence the quality of economic evaluations.

Our review found some key drivers of cost-effectiveness that are not always discussed. First, medication adherence should be incorporated in upcoming economic evaluations. It was found that medication non-adherence places a significant cost burden on healthcare systems [21]. Second, the drug costs, whether generic or branded, would affect the cost-effectiveness.

There are some limitations of this systematic review that must be addressed. First, this review included only fully published studies, and we did not look at grey literature and excluded conference abstracts. Second, most of the studies adopted the health care system perspective rather than the societal perspective, which limits the generalizability of results. Third, comparing economic outcomes is difficult because of the variability in currencies and the health system involved in different countries.

## Conclusion

Although most studies concluded that AIs are cost-effective compared to tamoxifen in early-stage BC, these results are disputable because they did not consider the adherence, the side effect profile, and the subgroup analysis. However, the overall quality of the studies included was average according to the CHEERS checklist. Characterizing heterogeneity should be considered in future studies.

## Electronic supplementary material

Below is the link to the electronic supplementary material.

Supplementary Material 1

## Data Availability

The datasets used and/or analyzed during this study are available from the corresponding author upon reasonable request.

## List of abbreviations

BC: Breast cancer
AIs: Aromatase inhibitors
CHEERS: Consolidated Health Economic Evaluation Reporting Standards
QALYs: Quality Adjusted Life Years
LYS: Life years saved
CEA: Cost- effectiveness analysis
CUA: Cost utility analysis
CBA: Cost-benefit analysis
ICER: Incremental cost-effectiveness ratio
RCT: Randomized controlled trials
SA: Sensitivity analysis
PSA: Probabilistic sensitivity analysis
