# Supplementary material for: Systematic review of economic evaluations of aromatase inhibitors in estrogen receptor-positive breast cancer: quality evaluation
Source: BMC Health Serv Res. 2023 Jun 26;23:689. doi: 10.1186/s12913-023-09432-5 (PMC10294338; doi:10.1186/s12913-023-09432-5)
Supplement: Supplementary file 1 — Supplementary Material 1 [file 12913_2023_9432_MOESM1_ESM.docx]

**Appendix S1:**

Search strategies:

Database: Ovid MEDLINE(R) and In-Process & Other Non-Indexed Citations <1946 to July 16, 2021>

Search Strategy:

--------------------------------------------------------------------------------

1 exp Breast Neoplasms/ (308448)

2 exp Breast/ or exp Breast Diseases/ (343824)

3 exp Neoplasms/ (3500221)

4 2 and 3 (312787)

5 (cancer* adj3 breast*).tw. (305831)

6 (neoplas* adj3 breast*).tw. (2906)

7 (carcinoma* adj3 breast*).tw. (42078)

8 (adenocarcinoma* adj3 breast*).tw. (3970)

9 (tumour* adj3 breast*).tw. (5833)

10 (tumor* adj3 breast*).tw. (32467)

11 (malignan* adj3 breast*).tw. (9989)

12 5 or 6 or 7 or 8 or 9 or 10 or 11 (344087)

13 1 or 4 or 12 (416249)

14 exp Aromatase Inhibitors/ (9211)

15 aromatase inhibitor*.tw. (7620)

16 (Nolvadex or Soltamox or Letrozole or Femara or Exemestane or Aromasin or anastrozole or Arimidex).tw. (5081)

17 14 or 15 or 16 (13185)

18 13 and 17 (8051)

19 exp Tamoxifen/ (21723)

20 (Tamoxifen or Novaldex or soltamox).tw. (23372)

21 19 or 20 (30331)

22 18 and 21 (3664)

23 Economics/ (27346)

24 exp "Costs and Cost Analysis"/ (247080)

25 Economics, Nursing/ (4005)

26 Economics, Medical/ (9138)

27 Economics, Pharmaceutical/ (2998)

28 exp Economics, Hospital/ (25197)

29 Economics, Dental/ (1918)

30 exp "Fees and Charges"/ (30795)

31 exp Budgets/ (13851)

32 budget*.ti,ab,kf. (231134)

33 (economic* or cost or costs or costly or costing or price or prices or pricing or pharmacoeconomic* or pharmaco-economic* or expenditure or expenditures or expense or expenses or financial or finance or finances or financed).ti,kf. (240609)

34 (economic* or cost or costs or costly or costing or price or prices or pricing or pharmacoeconomic* or pharmaco-economic* or expenditure or expenditures or expense or expenses or financial or finance or finances or financed).ab. /freq=2 (310574)

35 (cost* adj2 (effective* or utilit* or benefit* or minimi* or analy* or outcome or outcomes)).ab,kf. (172936)

36 (value adj2 (money or monetary)).ti,ab,kf. (2529)

37 exp models, economic/ (15700)

38 economic model*.ab,kf. (3486)

39 markov chains/ (15091)

40 markov.ti,ab,kf. (23987)

41 monte carlo method/ (29857)

42 monte carlo.ti,ab,kf. (51794)

43 exp Decision Theory/ (12511)

44 (decision* adj2 (tree* or analy* or model*)).ti,ab,kf. (26876)

45 or/23-44 (768968)

46 22 and 45 (152)

Database: Embase <1980 to 2020 July 16 >

Search Strategy:

--------------------------------------------------------------------------------

1 exp breast tumor/ (555899)

2 exp BREAST/ or exp BREAST DISEASE/ (638516)

3 exp neoplasm/ (4576727)

4 (cancer* adj3 breast*).tw. (444077)

5 (neoplas* adj3 breast*).tw. (3396)

6 (carcinoma* adj3 breast*).tw. (51904)

7 (adenocarcinoma* adj3 breast*).tw. (5320)

8 (tumour* adj3 breast*).tw. (8329)

9 (tumor* adj3 breast*).tw. (46539)

10 (malignan* adj3 breast*).tw. (13849)

11 5 or 6 or 7 or 8 or 9 or 10 (114654)

12 2 and 3 (580096)

13 1 or 11 or 12 (593571)

14 exp aromatase inhibitor/ (33663)

15 aromatase inhibitor*.tw. (12522)

16 (Nolvadex or Soltamox or Letrozole or Femara or Exemestane or Aromasin or anastrozole or arimidex).tw. (13333)

17 14 or 15 or 16 (40569)

18 13 and 17 (22892)

19 exp Tamoxifen/ (64640)

20 (Tamoxifen or Novaldex or soltamox).tw. (35004)

21 19 or 20 (68682)

22 18 and 21 (13159)

23 *economics/ (26388)

24 exp *"costs and cost analysis"/ (77832)

25 (economic adj2 model*).mp. (8277)

26 (cost minimi* or cost-utilit* or health utilit* or economic evaluation* or economic review* or cost outcome or cost analys?s or economic analys?s or budget* impact analys?s).ti,ab,kw. (55161)

27 (cost-effective* or pharmacoeconomic* or pharmaco-economic* or cost-benefit or costs).ti,kw. (113239)

28 (life year or life years or qaly* or cost-benefit analys?s or cost-effectiveness analys?s).ab,kw. (51504)

29 (cost or economic*).ti,kw. and (costs or cost-effectiveness or markov).ab. (98002)

30 or/23-29 (266180)

31 22 and 30 (253)

CRD database: (DARE, NHS EED and HTA):

|  | 1 | MeSH DESCRIPTOR Breast Neoplasms EXPLODE ALL TREES | 1798 |
| --- | --- | --- | --- |
|  | 2 | (aromatase inhibitor) | 38 |
|  | 3 | (anastrozole) OR (Arimidex) | 63 |
|  | 4 | (letrozole ) OR (femara) | 75 |
|  | 5 | (exemestane ) OR (aromasin) | 38 |
|  | 6 | (tamoxifen) OR (soltamox) OR (nolvadex ) | 235 |
|  | 7 | #2 OR #3 OR #4 OR #5 | 117 |
|  | 8 | #1 AND #7 | 89 |
|  | 9 | #6 AND #8 | 62 |

Scopus: (19/07/2021):

The search terms used were:

1. “breast cancer” or breast neoplasm or carcinoma or malignan* or sarcoma or tumo?r (444,406)
2. Search within results: estrogen receptor positive (55,727)
3. Search within results: cost effective or pharmacoeconomic* or markov model or economic model or decision model (771)
4. Search within results: tamoxifen or nolvadix or soltamox (529)
5. Search within results: “aromatase inhibitor” or letrozole or femara or exemestane or aromasin or anastrozole or arimidex (267)

After deleting duplicate and screening titles and abstracts, I end up with 37 documents

Table S 1. Handling structural and methodological uncertainty.

| No. | Increased mortality following any adverse event? | Did the analysis address the following sub-groups? | | | | | | | |  |  |
| --- | --- | --- | --- | --- | --- | --- | --- | --- | --- | --- | --- |
|  |  | Older women? | Women at low risk of breast cancer recurrence? | Women at high risk of fracture? | Women with high risk of cardio-vascular disease? | Women at high risk of stroke? | Women at high risk of thrombo-embolism? | Women at high risk of endo-metrial cancer? | Women with multiple co-morbidities? | SA for extrapolating beyond the follow-up time of studies | SA for the discount rate |
|  |  |  |  |  |  |  |  |  |  |  |  |
| 1^10^ | √ | √ | x | x | x | x | x | x | x | x | √ |
| 2^11^ | √ | x | x | x | x | x | x | x | x | x | x |
| 3^12^ | x | √ | x | x | x | x | x | x | x | x | √ |
| 4^9^ | x | x | x | x | x | x | x | x | x | x | x |
| 5^14^ | x | x | x | x | x | x | x | x | x | x | x |
| 6^16^ | x | x | x | x | x | x | x | x | x | x | √ |
| 7^15^ | √ | x | x | x | x | x | x | x | x | √ | √ |
| 8^13^ | √ | x | x | x | x | x | x | x | x | √ | √ |

Table S2 Parameter Uncertainty

|  | Handling of parameter uncertainty | | | | | | | |
| --- | --- | --- | --- | --- | --- | --- | --- | --- |
|  | SA on risk of BC recurrence | SA on AE | | | | | PSA | VOI |
|  |  | Fracture | Cardiovascular events | Stroke | Thromboembolism | Endometrial cancer |  |  |
| 1^10^ | √ | √ | x | x | x | √ | √ | x |
| 2^11^ | √ | √ | √ | √ | √ | √ | √ | √ |
| 3^12^ | √ | √ | √ | x | √ | √ | x | x |
| 4^9^ | √ | x | x | x | x | x | √ | x |
| 5^14^ | √ | √ | x | x | √ | x | √ | x |
| 6^16^ | √ | x | x | x | x | x | √ | x |
| 7^15^ | √ | √ | √ | x | √ | √ | x | x |
| 8^13^ | √ | √ | √ | √ | √ | √ | √ | x |

**Supporting information**

Appendix S1:

Complete search strategy

Table S1:

Handling structural and methodological uncertainty.

Table S2:

Handling parameter uncertainty
